# Increase of reactive oxygen species by desferrioxamine during experimental Chagas' disease

**DOI:** 10.1179/174329210X12650506623528

**Published:** 2013-07-19

**Authors:** Amanda Fortes Francisco, Paula Melo de Abreu Vieira, Jerusa Marilda Arantes, Maisa Silva, Maria Lúcia Pedrosa, Silvana Maria Elói-Santos, Olindo Assis Martins-Filho, Andréa Teixeira-Carvalho, Márcio Sobreira Silva Araújo, Washington Luiz Tafuri, Cláudia Martins Carneiro

**Affiliations:** Laboratório de Imunopatologia, Núcleo de Pesquisas em Ciências Biológicas, Instituto de Ciências Exatas e Biológicas, Universidade Federal de Ouro Preto, Ouro Preto, Brazil; Laboratório de Biomarcadores de Diagnóstico e Monitoração, Centro de Pesquisas René Rachou, Fundação Osvaldo Cruz, Belo Horizonte, Brazil, Pós-Graduação em Patologia, Faculdade de Medicina, Universidade Federal de Minas Gerais, Belo Horizonte, Brazil; Laboratório de Bioquímica e Biologia Molecular, Núcleo de Pesquisas em Ciências Biológicas, Instituto de Ciências Exatas e Biológicas, Universidade Federal de Ouro Preto, Ouro Preto, Brazil; Departamento de Propedêutica Complementar, Faculdade de Medicina, Universidade Federal de Minas Gerais, Belo Horizonte, Brazil; Laboratório de Biomarcadores de Diagnóstico e Monitoração, Centro de Pesquisas René Rachou, Fundação Osvaldo Cruz, Belo Horizonte, Brazil; Laboratório de Imunopatologia (sala 40, ICEB II), Nucleo de Pesquisas em Ciências Biológicas (NUPEB), Instituto de Ciências Exatas e Biológicas (ICEB), Universidade Federal de Ouro Preto (UFOP), Campus Universitário Morro do Cruzeiro, 35400-000 Ouro Preto, MG, Brazil; Departamento de Análises Clinicas, Escola de Farmácia, Universidade Federal de Ouro Preto, Ouro Preto, Brazil. Email: carneirocm@gmail.com, carneiro@ef.ufop.br

**Keywords:** DESFERRIOXAMINE, OXIDATIVE STRESS, CHAGAS' DISEASE, TRYPANOSOMA CRUZI

## Abstract

Oxidative stress is common in inflammatory processes associated with many diseases including Chagas' disease. The aim of the present study was to evaluate, in a murine model, biomarkers of oxidative stress together with components of the antioxidant system in order to provide an overview of the mechanism of action of the iron chelator desferrioxamine (DFO). The study population comprised 48 male Swiss mice, half of which were treated daily by intraperitoneal injection of DFO over a 35-day period, while half were administered sterile water in a similar manner. On the 14th day of the experiment, 12 DFO-treated mice and an equal number of untreated mice were experimentally infected with *Trypanosoma cruzi*. Serum concentrations of nitric oxide and superoxide dismutase and hepatic levels of total glutathione, thiobarbituric acid reactive species and protein carbonyl, were determined on days 0, 7, 14 and 21 post-infection. The results obtained revealed that DFO enhances antioxidant activity in the host but also increases oxidative stress, indicating that the mode of action of the drug involves a positive contribution to the host together with an effect that is not beneficial to the parasite.


Research article

### Increase of reactive oxygen species by desferrioxamine during experimental Chagas' disease

**Amanda Fortes Francisco[1], Paula Melo de Abreu Vieira[1], Jerusa Marilda Arantes[3,4], Maisa Silva[2], Maria Lúcia Pedrosa[2], Silvana Maria Elói-Santos[5], Olindo Assis Martins-Filho[3], Andréa Teixeira-Carvalho[3], Márcio Sobreira Silva Araújo[3], Washington Luiz Tafuri[1], Cláudia Martins Carneiro[1,6]**

*[1]Laboratório de Imunopatologia and [2]Laboratório de Bioquímica e Biologia Molecular, Núcleo de Pesquisas em Ciências Biológicas, Instituto de Ciências Exatas e Biológicas, Universidade Federal de Ouro Preto, Ouro Preto, Brazil*
*[3]Laboratório de Biomarcadores de Diagnóstico e Monitoração, Centro de Pesquisas René Rachou, Fundação Osvaldo Cruz, Belo Horizonte, Brazil*
*[4]Pós-Graduação em Patologia, and [5]Departamento de Propedêutica Complementar, Faculdade de Medicina, Universidade Federal de Minas Gerais, Belo Horizonte, Brazil*
*[6]Departamento de Análises Clinicas, Escola de Farmácia, Universidade Federal de Ouro Preto, Ouro Preto, Brazil*

Oxidative stress is common in inflammatory processes associated with many diseases including Chagas' disease. The aim of the present study was to evaluate, in a murine model, biomarkers of oxidative stress together with components of the antioxidant system in order to provide an overview of the mechanism of action of the iron chelator desferrioxamine (DFO). The study population comprised 48 male Swiss mice, half of which were treated daily by intraperitoneal injection of DFO over a 35-day period, while half were administered sterile water in a similar manner. On the 14th day of the experiment, 12 DFO-treated mice and an equal number of untreated mice were experimentally infected with *Trypanosoma cruzi*. Serum concentrations of nitric oxide and superoxide dismutase and hepatic levels of total glutathione, thiobarbituric acid reactive species and protein carbonyl, were determined on days 0, 7, 14 and 21 post-infection. The results obtained revealed that DFO enhances antioxidant activity in the host but also increases oxidative stress, indicating that the mode of action of the drug involves a positive contribution to the host together with an effect that is not beneficial to the parasite.

**Keywords:** desferrioxamine, oxidative stress, Chagas' disease, *Trypanosoma cruzi*

---

*Abbreviations*: DFO, desferrioxamine; dpi, days post-infection; GPx, glutathione peroxidase; GSH, reduced glutathione; GSSG, glutathione disulfide; GT, total glutathione; INT, infected with *T. cruzi* but not treated with DFO; IP, intraperitoneal; IT, infected with *T. cruzi* and treated with DFO; NIT, not infected with *T. cruzi* but treated with DFO; NINT, not infected with *T. cruzi* and not treated with DFO; NO, nitric oxide; NOS, nitric oxide synthase; PC, carbonyl protein; ROS, reactive oxygen species; SOD, superoxide dismutase; TBARS, thiobarbituric acid reactive substances; TNB, 5-thio-2-nitrobenzoic acid.

---

Correspondence to: Dr C.M. Carneiro, Laboratório de Imunopatologia (sala 40, ICEB II), Nucleo de Pesquisas em Ciências Biológicas (NUPEB), Instituto de Ciências Exatas e Biológicas (ICEB), Universidade Federal de Ouro Preto (UFOP), Campus Universitário Morro do Cruzeiro, 35400-000 Ouro Preto, MG, Brazil. Tel: +55 31 35591694; Fax: +55 31 35591680; E-mail: carneirocm@gmail.com or carneiro@ef.ufop.br
Received 13 February 2010, revised manuscript accepted 18 May 2010

**© W. S. Maney and Son Ltd 2010**
**DOI 10.1179/174329210X12650506623528**
**MORE OpenChoice articles are open access and distributed under the terms of the Creative Commons Attribution License 3.0**

### Introduction

Chagas' Disease continues to pose a serious threat to human health in much of Latin America, and has become the most important emerging parasitic disease in developed countries. There is growing evidence to suggest that during the course of infection by *Trypanosoma cruzi*, the myocardium is exposed to injuries induced by sustained oxidative stress, and that these may contribute to disease progression. The principal causes of oxidative stress are reactive oxygen species (ROS), which may be broadly defined as derivatives of molecular oxygen, *e.g.* $O_2^{\bullet-}$, $HO^{\bullet}$ and hydrogen peroxide. ROS are unstable and react rapidly with other free radicals and macromolecules in chain reactions to generate increasingly harmful oxidants.[1] Acute *T. cruzi* infection induces $H_2O_2$ release by peripheral blood monocytes and spleen macrophages in rats infected with the Y strain.[2] During the course of development of Chagas' disease, high levels of ROS can be produced as a consequence of tissue destruction caused by toxic secretions from the parasite, immune-mediated cytotoxic reactions and secondary damage to mitochondria.[1]

All aerobic organisms are continuously subjected to ROS generated by oxidative metabolism, the detoxification of xenobiotics or the action of ultraviolet radiation and several other mechanisms, because anaerobic organisms do not need such antioxidant adaptations, and there are many other mechanisms that generate ROS in animals, fungi, algae, protists and plants. In response, organisms have developed a variety of antioxidant defense systems to cope with ROS, although it is important to note that the defense strategies of parasitic protozoa exhibit major differences one from another and also in comparison with their mammalian hosts. Many antioxidant defense strategies depend on the action of antioxidant enzymes, including superoxide dismutase (SOD), glutathione peroxidase (GPx) and catalase, or low molecular weight antioxidants due to their importance in ROS (hydrogen peroxide) detoxification[3]. SOD catalyses the conversion of the superoxide anion into hydrogen peroxide and oxygen, and exists in a number of different isoforms. Unlike other eukaryotic parasites, the trypanosomatids (*Leishmania* spp. and *Trypanosoma* spp.) together with *Plasmodium* spp. have an iron-containing SOD isoform that is normally found only in bacteria.[4,5] GPx exists in five isoforms and catalyses reduction by glutathione of hydrogen peroxide or alkyl peroxide to water or alcohol, respectively.[1]

A number of assays are available that enable assessment of the level of oxidative stress induced by different biological agents within an organism. The determination of lipid peroxidation in terms of thiobarbituric acid reactive substances (TBARS) is considered to be a very useful, cheap and easy assay for evaluating oxidative stress. However, this assay, despite common use in evaluating oxidative stress, has some limitations, especially when measured by spectrophotometry and not by fluorescence or HPLC. Additionally, the direct attack of protein by ROS leads to the formation of carbonyl groups, such that the estimation of protein carbonyl (PC) provides an additional (or complementary) measure of the effect of oxidative stress.

Desferrioxamine (DFO; Desferal®, Novartis, Basel, Switzerland) is a hexadentate iron chelator that complexes with iron in a 1:1 molar ratio to yield the stable complex ferrioxamine (stability constant 1031).[6] DFO is believed to access the pools of iron in the liver and in the reticulo-endothelial system that are together responsible for the major proportion of the body iron burden. However, since DFO permeates into cells relatively slowly, because of its large molecular size and hydrophilic nature, its efficiency as an intracellular chelator is limited.[5,7] Various studies[2,8–11] have established that administration of DFO in *T. cruzi* infection modifies the course of Chagas' disease, reducing parasitemia and mortality. Results obtained using a murine model have shown, however, that whilst there were reductions in the number of circulating parasites and in mortality, the animals did not suffer from anemia.[8,9] It was concluded, therefore, that DFO had a direct effect on the parasite and not on the reduction of iron supply in the host.

The aim of the present study was to evaluate the relationship between oxidative stress (assessed by TBARS, PC and nitrate/nitrite assays) and antioxidant defenses (evaluated in terms of SOD and GPx levels) in *T. cruzi*-infected mice that had been treated with DFO in order to clarify the mode of action of the drug.

### Materials and methods

Details of the project were submitted to, and approved by, the Ethical Committee on Animal Research of the Universidade Federal de Ouro Preto and by the institutional Animal Care and Use Committee (CETEA 153/07) of the Universidade Federal de Minas Gerais. All procedures were carried out in compliance with current Brazilian regulations relating to Experimental Biology and Medicine as described in the guidelines issued by the Colégio Brasileiro de Experimentaçao Animal (COBEA) in 2006.

Experimental animals comprised 48 male Swiss mice, each approximately 30 days old. Throughout the 35-day period of the study, animals received a commercial diet in pellet form (Nuvilab CR1) together with sterilized water *ad libitum*. Animals were distributed randomly into four equal groups ($n$ = 12) as follows: control animals (NINT group) were not infected with *T. cruzi* and were not treated with DFO; group INT animals were infected with *T. cruzi* but were not treated with DFO; group NIT animals were not infected with *T. cruzi* but were treated with DFO; and group IT animals were infected with *T. cruzi* and treated with DFO. Animals belonging to the treatment groups received daily doses of DFO (5 mg; 0.05 ml) by intraperitoneal (IP) injection for 14 days prior to infection and for a further 21 days post-infection (dpi).

Experimental animals in the NINT and INT groups were submitted to the same conditions of stress as the treated animals by daily administration of sterile water (0.05 ml) by IP injection. On day 14 of the study period, the 24 animals comprising IT and INT groups were infected by IP injection of 500 blood-stream forms of *T. cruzi* Y strain,[12] which is considered to be partially resistant to chemotherapeutic treatment.[5,13]

Peripheral blood samples were collected from the orbital plexus of animals at 0, 7, 14 and 21 dpi. In each case, the serum was separated and stored frozen (–70ºC) until required for nitrate/nitrite and SOD analysis. In order to determine the levels of GT, TBARS and PC in hepatocytes, 12 animals in each group were sacrificed by cervical dislocation at 0, 7, 14 and 21 dpi, their livers were removed and immediately frozen to –70ºC and stored at this temperature until required for analysis.

### *Assay of antioxidant enzymes*

SOD activity was quantified using Fluka (St Louis, MO, USA) SOD determination kit (catalogue #19160) based on the capacity of the antioxidant enzyme to inhibit the reduction of 2-(4-iodophenyl)-3-(4-nitrophenyl)-5-(2,4-disulfophenyl)-2H-tetrazolium, monosodium salt by superoxide anions (generated *in situ* by a xanthine/xanthine oxidase system) to yield the corresponding formazan dye ($\lambda_{max}$ 450 nm).

Total glutathione (GT), *i.e.* reduced glutathione (GSH) and glutathione disulfide (GSSG), was assayed using a kinetic method (Sigma, St Louis, MO, USA; product code CS0260) in which GSH reduced 5,5'-dithiobis(2-nitrobenzoic acid) to 5-thio-2-nitrobenzoic acid (TNB) and GSSG, whilst the latter was recycled *in situ* by glutathione reductase and NADPH. TNB was determined spectrophotometrically at 412 nm.

### *Assay of protein carbonyls*

The carbonyl moieties formed by the oxidation of protein by ROS were assayed using a conventional spectrophotometric procedure based on reaction with 2,4-dinitrophenyl hydrazine to form the corresponding hydrazone derivatives. Determination of serum PC concentration was carried out according to a published procedure,[14] whilst total proteins were assayed according to the method of Lowry *et al.*[15] PC was determined spectrophotometrically at 370 nm.

### *Determination of lipid peroxidation*

Determination of TBARS concentration was based on the ability of the thiobarbituric acid to bind to oxidized lipids, this dosage was performed as described by Buege and Aust.[16] TBARS were determined spectrophotometrically at 535 nm.

### *Determination of serum nitrate/nitrite*

Levels of endogenously indirect production of NO were measured by nitrate/nitrite concentrations assessed by reducing serum nitrate to nitrite with nitrate reductase (1 U/ml) followed by the Griess reaction.[17] Nitrite concentrations were determined by extrapolation from standard curves, constructed using various concentrations of sodium nitrite, and the results were expressed as micromolar. Nitrate/nitrite concentrations was measured by an ELISA plaque (Molecular Devices, E Max, USA) at 570 nm.

### *Statistical analysis*

Between-group differences (NINT × NIT, INT × IT, NINT × INT and NIT × IT) in the levels of SOD, GT, nitrate/nitrite, TBARS and PC and between days after infection were analyzed by one-way analysis of variance and Tukey post-tests. In all cases, differences were considered statistically significant when $P$ values were $< 0.05$.

### Results

No differences between the experimental and control groups were observed in the levels of nitrate/nitrite at 0 and 7 dpi, PC at 0, 14 and 21 dpi and TBARS at 0 and 14 dpi. A significant increase in the level of TBARS in the INT group compared with the control (NINT group) was observed at 7 dpi (Fig. 1A), whilst at 21 dpi TBARS production in IT group animals was significantly increased compared with the NIT and INT groups (Fig. 1C).

Figure 1B reveals that at 7 dpi, treatment with DFO had induced a significant increase in the levels of PC in the IT group compared with the NIT group. Significant increases in serum nitrate/nitrite levels were also observed at 21 dpi in animals of the IT

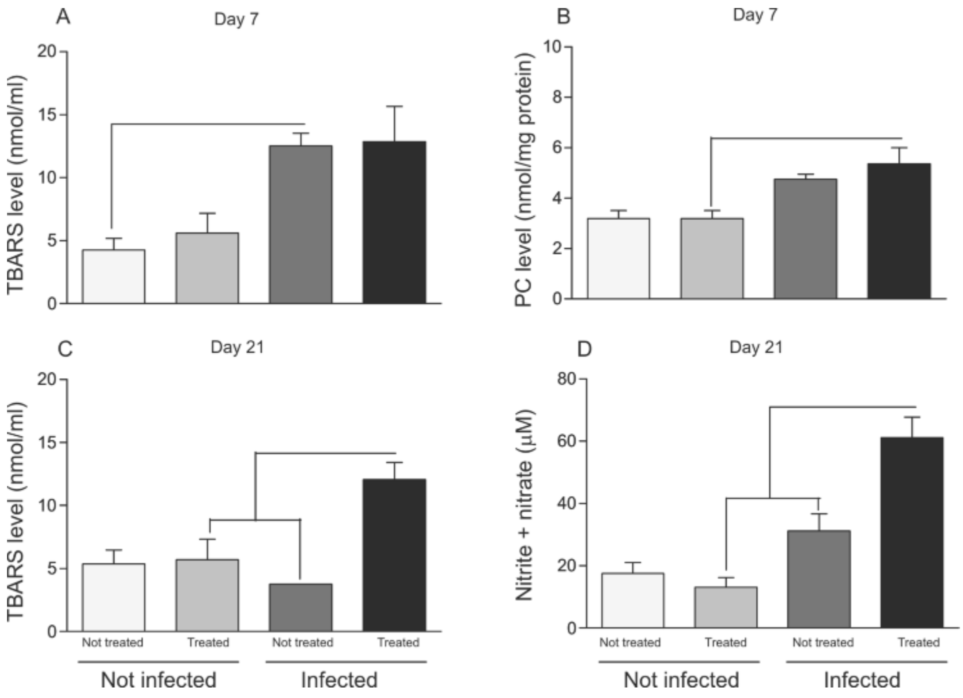

**Figure 1 TBARS and PC levels in hepatocytes and serum levels of nitrate/nitrite in mice not infected or infected with *T. cruzi* and not treated or treated with desferrioxamine at days 7 and 21. Significant differences ($P < 0.05$) between groups are indicated by connecting lines**

group when compared with the NIT and INT groups (Fig. 1D). Figure 2A reveals that at 21 dpi, treatment with DFO in animals of IT group had induced a significant increase in serum nitrate/nitrite levels compared with 0, 7 and 14 dpi. Additionally, serum nitrate/nitrite levels in infected but untreated mice (INT group) were significantly higher at 14 dpi than those of the control animals (NINT group; data not shown). Figure 2B reveals that at 21 dpi, treatment with DFO in animals of IT group had induced a significant increase in SOD activity compared with 0, 7 and 14 dpi.

No differences between the experimental and control groups were observed in the levels of GT at 0, 7 and 21 dpi or in SOD activity at 0, 7 and 14 dpi (data not shown). At 14 dpi, GT production in the livers of *T. cruzi*-infected mice that had been treated with DFO (IT group) was reduced significantly in comparison with non-infected but treated animals (NIT group) as shown in Figure 3A.

At 21 dpi, SOD activity in serum of mice belonging to the IT group was increased significantly compared with those of NIT and INT group animals, indicating an antioxidant effect of DFO when associated with infection (Fig. 3B).

No differences at 0, 7, 14 and 21 dpi were observed in animals of the NINT, NIT and INT groups (data not shown).

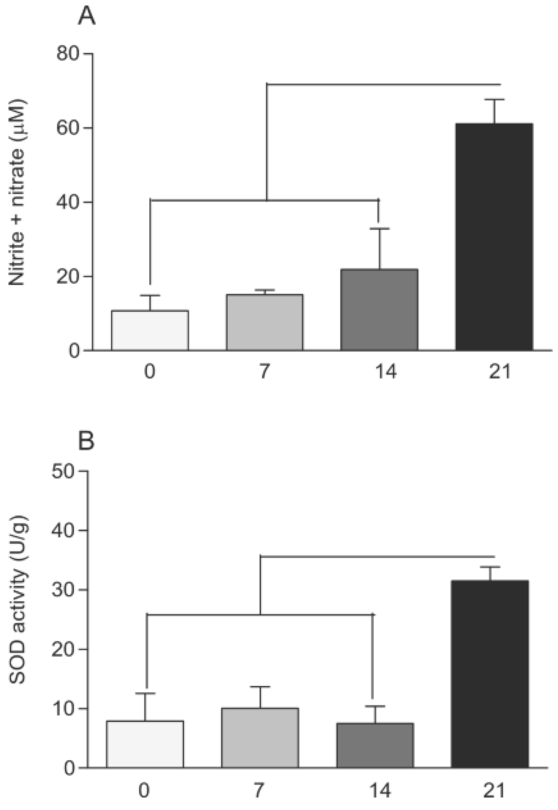

**Figure 2 Serum levels of nitrate/nitrite and serum activities of SOD in mice infected with *T. cruzi* and treated with desferrioxamine at days 0, 7, 14 and 21. Significant differences ($P < 0.05$) between days are indicated by connecting lines**

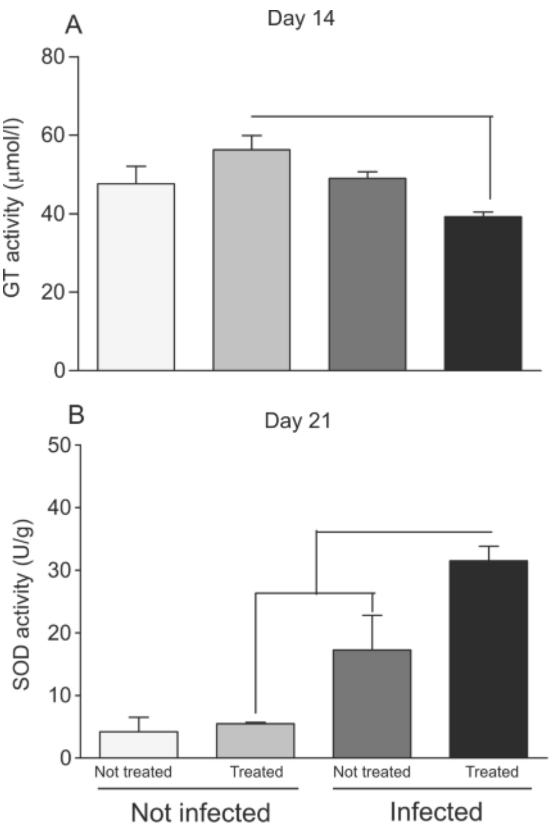

**Figure 3 GT levels in hepatocytes and serum activities of SOD in mice not infected or infected with *T. cruzi* and not treated or treated with desferrioxamine at days 14 and 21. Significant differences ($P < 0.05$) between groups are indicated by connecting lines**

### Discussion

In the present study, levels of PC were elevated in the IT group (Fig. 1B) suggesting that infection and DFO together increased oxidative stress in the host in order to exert some damage on the parasite. Additionally, the increases in production of TBARS in animals of the INT group that were observed at 7 dpi (Fig. 1A) indicate that infection initially augmented these reactive species. It must be pointed out that different organs respond in different ways in relation to ROS production, and that a high degree of oxidative stress has been detected in the peripheral blood of chagasic mice.[5] However, treatment with DFO promoted an increase in oxidative stress over a longer period of time (*i.e.* up to 21 dpi), suggesting that this may represent a possible mechanism in the reduction of parasitemia.

Nitric oxide (NO) is known to exert microbicidal or microbiostatic effects against many micro-organisms.[3] Gradual increases in the levels of nitrate/nitrite in animals of the IT group occurred throughout the post-infection treatment period (Fig. 1D) indicating that the action of DFO must be associated with infection. The increase of nitrate/nitrite in the INT group of animals (data not shown) was expected since *T. cruzi* infection stimulates the production of tumor necrosis factor-$\alpha$ and interferon-$\gamma$, and this culminates in the increased expression of inducible nitric oxide synthase (iNOS) and the subsequent production of NO by macrophages.[18] Although details of the mechanism by which iNOS is activated are unclear, infection typically increases NO production by 30%.[19]

The decrease in hepatic GT concentration in infected animals that had been treated with DFO (Fig. 3A) indicates the ability of the drug to increase the defense of the host against oxidative stress associated infection. Since GT levels in the NIT group were unchanged in comparison with both untreated groups, it is likely that DFO up-regulates GPx only in the presence of *T. cruzi* infection. Experimental studies have shown that host response to acute infection by *T. cruzi* involves the up-regulation of the glutathione antioxidant defense system comprising GPx, GSH and oxidized forms of glutathione.[1] However, during the progression of Chagas' disease in mice, glutathione defense becomes non-responsive to chronic oxidative stress and cardiac levels of GPx are reduced; a systemic GPx and SOD down-regulation is also observed in mice and humans under chronic conditions.[20,21] It is important to note that trypanosomatids do not possess the selenium-dependent glutathione peroxidase/glutathione reductase system found in other cells.[22]

In the present study, the levels of SOD in animals of the IT group occurred throughout the post-infection treatment period (Fig. 3B) indicating that the action of DFO must be associated with infection. In this respect, the infection-related effect of DFO is important in increasing the level of the antioxidant enzyme since SOD represents the first line of defense against ROS. It has previously been established that SOD activity in the erythrocytes of patients with mild cardiac impairment was higher than that found in controls, but no differences were detected in SOD activity in different chagasic groups.[21]

In terms of the glutathione and SOD defense systems, it is clear that DFO exerts an antioxidant action on the hepatocytes of the host that contributes to the protection against oxidative damage caused by Chagas' disease. Moreover, it would appear that the effects of DFO are apparent at the start of the parasitemia peak, leading to a reduction in the parasitemia curve of *T. cruzi* in DFO-treated animals as has been shown previously.[8,9] It is also possible that DFO causes an increase in the generation of ROS

through Fenton-type mechanisms since iron is an essential catalytic agent in such reactions. The use of this chelator may provoke a release of iron from ferritin as 'free iron' that was used in this reaction. It must be concluded, therefore, that the action of DFO increases the protection of the host against infection and also acts directly on the parasite itself, thus explaining the reductions in parasitemia and the rate of mortality in DFO-treated animals that have been reported earlier.[8,9] However, DFO also delayed increased production of nitrate/nitrite until 21 dpi, and this could be the reason why mortalities of infected animals treated with DFO have been observed by our group.[9]

### Conclusions

On the basis of the results presented herein, it is concluded that DFO possesses the capacity to increase antioxidant defense systems in the host, particularly by up-regulating SOD, as well as promoting a measured increase in oxidative stress that exerts pressure on the parasite. This suggests that DFO offers a balance of effects with respect to its capacity to promote protection in the host. The differences between the parasite and host in terms of the biochemical systems that afford the antioxidant defenses of the two organisms could provide exploitable targets for the development of an effective chemotherapy for Chagas' disease.

### Acknowledgements

This work was supported by the Coordenaçao de Aperfeiçoamento de Pessoal de Nivel Superior (CAPES), Centro de Pesquisas René Rachou, Fundação Osvaldo Cruz – FIOCRUZ (CPqRR), Fundação de Amparo a Pesquisa do Estado de Minas Gerais (FAPEMIG), Rede Mineira de Biotérios (RMB) and by the Universidade Federal de Ouro Preto (UFOP).

### References

1. Gupta S, Wen JJ, Garg NJ. Oxidative stress in Chagas' Disease. *Interdiscip Perspect Infect Dis* 2009; 2009: 190354.
2. Pedrosa ML, Silva ME, Silva ME, Silva MEC, Nicoli JR, Vieira EC. The effect of iron deficiency and iron overload on the evolution of Chagas' disease produced by three strains of *Trypanosoma cruzi* in CFW mice. *Comp Biochem Physiol A* 1990; **97**: 235–243.
3. De Groote MA, Fang FC. NO inhibitions: antimicrobial properties of nitric oxide. *Clin Infect Dis* 1995; **21 (Suppl. 2)**: S162–S165.
4. Bécuwe P, Gratepanche S, Fourmaux MN *et al*. Characterization of iron-dependent endogenous superoxide dismutase of *Plasmodium falciparum*. *Mol Biochem Parasitol* 1996; **76**: 125–134.
5. Zannineli G, Glickstein H, Breuer W, Brissot P, Hider RC, Cabantchik ZI. Chelation and mobilization of cellular iron by different classes of chelators. *Mol Pharmacol* 1997; **51**: 842–852.
6. Kushner JP, Porter JP, Olivieri NF. Secondary iron overload. *Hematol Am Soc Hematol Educ Program* 2001; **1**: 47–61.
7. Keberle H. The biochemistry of desferrioxamine and its relation to iron metabolism. *Ann NY Acad Sci* 1964; **119**: 758–768.
8. Arantes JM, Pedrosa ML, Martins HR *et al*. *Trypanosoma cruzi*: treatment with the iron chelator desferrioxamine reduces parasitemia and mortality in experimentally infected mice. *Exp Parasitol* 2007; **117**: 43–50.
9. Francisco AF, Vieira PMA, Arantes JM *et al*. *Trypanosoma cruzi*: effect of benznidazole therapy combined with the iron chelator desferrioxamine in infected mice. *Exp Parasitol* 2008; **120**: 314–319.
10. Lalonde RG, Holbein BE. Role of iron in *Trypanosoma cruzi* infection of mice. *J Clin Invest* 1984; **3**: 470–476.
11. Loo VG, Lalonde RG. Role of iron in intracellular growth of *Trypanosoma cruzi*. *Infect Immun* 1984; **45**: 726–730.
12. Silva LHP, Nussenzweig V. Sobre uma cepa altamente virulenta para o camundongo branco. *Folia Clin Biol* 1953; **20**: 191–203.
13. Filardi LS, Brener Z. Susceptibility and natural resistance of *Trypanosoma cruzi* strains to drugs used clinically in Chagas' Disease. *Trans R Soc Trop Med Hyg* 1987; **81**: 755–759.
14. Levine RL, Garland D, Oliver CN *et al*. Determination of carbonyl content in oxidatively modified proteins. *Methods Enzymol* 1990; **186**: 464–478.
15. Lowry OH, Rosebrough NJ, Farr AL, Randall RJ. Protein measurement with the Folin phenol reagent. *J Biol Chem* 1951; **193**: 265–275.
16. Buege JA, Aust SD. Microsomal lipid peroxidation. *Methods Enzymol* 1978; **52**: 302–310.
17. Green LC, Wagner DA, Gloglowski J, Skipper PL, Wishnok JS, Tannenbaum SR. Analysis of nitrate, nitrite, and ($^{15}$N)-nitrate in biological fluids. *Anal Biochem* 1982; **126**: 131–138.
18. MacMicking J, Xie QW, Nathan C. Nitric oxide and macrophage function. *Annu Rev Immunol* 1997; **5**: 323–350.
19. Philipp S, Cui L, Ludolph B, Kelm M, Schulz R, Cohen MV, Downey JM. Desferoxamine and ethyl-3,4-dihydroxybenzoate protect myocardium by activating noxidative stress and generating mitochondrial ROS. *Am J Physiol* 2006; **290**: H450–H457.
20. Pérez-Fuentes R, Torres-Rasgado E, Salgado-Rosas H, Zamora-Ginez I, Sánchez-Guillén MC. The anti-oxidant defence response in individuals with the indeterminate form of Chagas' disease (American trypanosomiasis). *Ann Trop Med Parasitol* 2008; **102**: 189–197.
21. De Oliveira TB, Pedrosa RC, Wilhelm Filho D. Oxidative stress in chronic cardiopathy associated with Chagas' disease. *Int J Cardiol* 2007; **116**: 357–363.
22. Fairlamb AH, Cerami A. Metabolism and functions of trypanothione in the kinetoplastida. *Annu Rev Microbiol* 1992; **46**: 695–729.

## References

[1] Gupta S, Wen JJ, Garg NJ Oxidative stress in Chagas' Disease. Interdiscip Perspect Infect Dis 2009; 2009: 190354. 19547716 10.1155/2009/190354 PMC2696642

[2] Pedrosa ML, Silva ME, Silva ME, Silva MEC, Nicoli JR, Vieira EC The effect of iron deficiency and iron overload on the evolution of Chagas' disease produced by three strains of Trypanosoma cruzi in CFW mice. Comp Biochem Physiol A 1990; 97: 235–243. 1710956 10.1016/0300-9629(90)90178-u

[3] De Groote MA, Fang FC NO inhibitions: antimicrobial properties of nitric oxide. Clin Infect Dis 1995; 21 (Suppl. 2): S162-S165. 8845445 10.1093/clinids/21.supplement_2.s162

[4] Bécuwe P, Gratepanche S, Fourmaux MN et al. Characterization of iron-dependent endogenous superoxide dismutase of Plasmodium fakiparum. Mol Biochem Parasitol 1996; 76: 125-134. 10.1016/0166-6851(95)02552-9 8920001

[5] Zannineli G, Glickstein H, Breuer W, Brissot P, Hider RC, Cabantchik ZI Chelation and mobilization of cellular iron by different classes of chelators. Mol Pharmaco11997; 51: 842-852. 10.1124/mol.51.5.842 9145923

[6] Kushner JP, Porter JP, Olivieri NF Secondary iron overload. Hematol Am Soc Hematol Educ Program 2001; 1: 47–61. 10.1182/asheducation-2001.1.47 11722978

[7] Keberle H The biochemistry of desferrioxamine and its relation to iron metabolism. Ann NY Acad Sci 1964; 119: 758–768. 14219455 10.1111/j.1749-6632.1965.tb54077.x

[8] Arantes JM, Pedrosa ML, Martins HR et al. Trypanosoma cruzi: treatment with the iron chelator desferrioxamine reduces parasitemia and mortality in experimentally infected mice. Exp Parasitol 2007; 117: 43–50. 17521632 10.1016/j.exppara.2007.03.006

[9] Francisco AF, Vieira PMA, Arantes JM et al. Trypanosoma cruzi: effect of benznidazole therapy combined with the iron chelator desferrioxamine in infected mice. Exp Parasitol 2008; 120: 314–319. 18789321 10.1016/j.exppara.2008.08.002

[10] Lalonde RG, Holbein BE Role of iron in Trypanosoma cruzi infection of mice. J Clin Invest 1984; 3: 470–476. 10.1172/JCI111233 PMC425038 6421877

[11] Loo VG, Lalonde RG Role of iron in intracellular growth of Trypanosoma cruzi. Infect Immun 1984; 45: 726–730. 6381312 10.1128/iai.45.3.726-730.1984 PMC263357

[12] Silva LHP, Nussenzweig V Sobre uma cepa altamente virulenta para o camundongo branco. Folia Clin Biol 1953; 20: 191–203.

[13] Filardi LS, Brener Z Susceptibility and natural resistance of Trypanosoma cruzi strains to drugs used clinically in Chagas' Disease. Trans R Soc Trop Med Hyg 1987; 81: 755–759. 3130683 10.1016/0035-9203(87)90020-4

[14] Levine RL, Garland D, Oliver CN et al. Determination of carbonyl content in oxidatively modified proteins. Methods Enzymol 1990; 186: 464–478. 1978225 10.1016/0076-6879(90)86141-h

[15] Lowry OH, Rosebrough NJ, Farr AL, Randall RJ Protein measurement with the Folin phenol reagent. J Biol Chem 1951; 193: 265–275. 14907713

[16] Buege JA, Aust SD Microsomal lipid peroxidation. Methods Enzymol 1978; 52: 302–310. 672633 10.1016/s0076-6879(78)52032-6

[17] Green LC, Wagner DA, Gloglowski J, Skipper PL, Wishnok JS, Tannenbaum SR Analysis of nitrate, nitrite, and (“N)-nitrate in biological fluids. Anal Biochem 1982; 126: 131–138. 7181105 10.1016/0003-2697(82)90118-x

[18] MacMicking J, Xie QW, Nathan C Nitric oxide and macrophage function. Annu Rev Immuno11997; 5: 323-350. 10.1146/annurev.immunol.15.1.323 9143691

[19] Philipp S, Cui L, Ludolph B, Kelm M, Schulz R, Cohen MV, Downey JM Desferoxamine and ethyl-3,4-dihydroxybenzoate protect myocardium by activating noxidative stress and generating mitochondrial ROS. Am J Physiol 2006; 290: H450–H457. 10.1152/ajpheart.00472.2005 16155105

[20] Pérez-Fuentes R, Torres-Rasgado E, Salgado-Rosas H, Zamora-Ginez I, Sánchez-Guillén MC The anti-oxidant defence response in individuals with the indeterminate form of Chagas' disease (American trypanosomiasis). Ann Trop Med Parasitol 2008; 102: 189–197. 18348773 10.1179/136485908X267858

[21] De Oliveira TB, Pedrosa RC, Wilhelm Filho D Oxidative stress in chronic cardiopathy associated with Chagas' disease. Int J Cardiol 2007; 116: 357–363. 16859784 10.1016/j.ijcard.2006.04.046

[22] Fairlamb AH, Cerami A Metabolism and functions of trypanothione in the kinetoplastida. Annu Rev Microbiol 1992; 46: 695–729. 1444271 10.1146/annurev.mi.46.100192.003403

